# Pan-London Network for Psychosis-Prevention (PNP)

**DOI:** 10.3389/fpsyt.2019.00707

**Published:** 2019-10-11

**Authors:** Paolo Fusar-Poli, Andrés Estradé, Tom J. Spencer, Susham Gupta, Silvia Murguia-Asensio, Savithasri Eranti, Kerry Wilding, Olivier Andlauer, Jonathan Buhagiar, Martin Smith, Sharon Fitzell, Victoria Sear, Adelaide Ademan, Andrea De Micheli, Philip McGuire

**Affiliations:** ^1^Early Psychosis: Interventions and Clinical-detection (EPIC) Lab, Department of Psychosis Studies, Institute of Psychiatry, Psychology & Neuroscience, King’s College London, London, United Kingdom; ^2^OASIS Service, South-London and Maudsley NHS Foundation Trust, London, United Kingdom; ^3^Department of Brain and Behavioral Sciences, University of Pavia, Pavia, Italy; ^4^National Institute for Health Research, Maudsley Biomedical Research Centre, South London and Maudsley NHS Foundation Trust, London, United Kingdom; ^5^Department of Clinical and Health Psychology, Catholic University, Montevideo, Uruguay; ^6^Department of Psychosis Studies, Institute of Psychiatry, Psychology and Neuroscience, King’s College London, London, United Kingdom; ^7^HEADS UP, East London NHS Foundation Trust, London, United Kingdom; ^8^THEDS, East London NHS Foundation Trust, London, United Kingdom; ^9^NEIS, East London NHS Foundation Trust, London, United Kingdom; ^10^Luton and Bedfordshire Service for the Prevention of Psychosis, East London NHS Foundation Trust, London, United Kingdom; ^11^Centre for Psychiatry, Wolfson Institute of Preventive Medicine, Barts and the London School of Medicine and Dentistry, Queen Mary University of London, London, United Kingdom

**Keywords:** psychosis, schizophrenia, risk, at risk mental state, structured interview for psychosis-risk syndromes, prevention

## Abstract

**Background:** The empirical success of the Clinical High Risk for Psychosis (CHR-P) paradigm is determined by the concurrent integration of efficient detection of cases at-risk, accurate prognosis, and effective preventive treatment within specialized clinical services. The characteristics of the CHR-P services are relatively under-investigated.

**Method:** A Pan-London Network for psychosis prevention (PNP) was created across urban CHR-P services. These services were surveyed to collect the following: description of the service and catchment area, outreach, service users, interventions, and outcomes. The results were analyzed with descriptive statistics and Kaplan Meier failure function.

**Results:** The PNP included five CHR-P services across two NHS Trusts: Outreach and Support In South-London (OASIS) in Lambeth and Southwark, OASIS in Croydon and Lewisham, Tower Hamlets Early Detection Service (THEDS), City & Hackney At-Risk Mental State Service (HEADS UP) and Newham Early Intervention Service (NEIS). The PNP serves a total population of 2,318,515 Londoners (830,889; age, 16–35 years), with a yearly recruitment capacity of 220 CHR-P individuals (age, 22.55 years). Standalone teams (OASIS and THEDS) are more established and successful than teams that share their resources with other mental health services (HEADS UP, NEIS). Characteristics of the catchment areas, outreach and service users, differ across PNP services; all of them offer psychotherapy to prevent psychosis. The PNP is supporting several CHR-P translational research projects.

**Conclusions:** The PNP is the largest CHR-P clinical network in the UK; it represents a reference benchmark for implementing detection, prognosis, and care in the real-world clinical routine, as well as for translating research innovations into practice.

## Introduction

Under standard care, outcomes of psychotic disorders are characterized by poor response to treatments, high chronicity and disability, low functional level, and high burden to families and society (1). Early interventions at the time of a first psychotic episode are associated with some important clinical benefits (2). However, these interventions are not particularly effective at preventing relapses (2) or reducing the duration of untreated psychosis (3). According to the definition of the World Health Organization, preventive strategies for mental disorders integrate the Gordon’s classification of the prevention of physical illness (universal—targeted at the general public, selective—targeted at those with risk factors or indicated targeted at those with minimal signs or symptoms of mental disorders) and the classic public health classification (primary—seeking to prevent the onset of a mental disorder, secondary—seeking to lower the rate of established disorder, tertiary—seeking to reduce disability and relapses) (4).

Universal, selective, and indicated preventive interventions are “included within primary prevention in the public health classification” [page 17 in (4)].

Preventive strategies have entered clinical psychiatry through the Clinical High Risk paradigm for Psychosis [CHR-P hereafter (5)]. The conceptual and operational framework that characterizes the CHR-P paradigm has been reviewed elsewhere (6, 7). Briefly, young (typically 14–35 years) individuals presenting with attenuated symptoms of psychosis coupled with help-seeking behavior (8) and functional impairments (9) are assessed through validated psychometric assessment interviews (10). These interviews ascertain in the context of a clinical assessment (11), whether the individual is at-risk of developing psychosis. The increased risk is accounted for by the accumulation of specific risk factors for psychosis (12) and cumulates to about 20% of CHR-P developing the disorder at 2-year follow-up [from eTable 4 in Fusar-Poli et al. (13)]. The individuals who will eventually meet the CHR-P intake criteria will then be offered specialized care which involves needs-based intervention and preventive treatments [for a recent review of their efficacy, see the studies by David et al. (14, 15)]. These treatments have a threefold aim of reducing the presenting problems, reducing the risk of progression from a CHR-P stage to the first onset of the disorder, and reducing the duration of untreated psychosis in the case the disorder would occur (16, 17). In line with these arguments, interventions in CHR-P individuals are defined as indicated primary prevention of psychosis. The empirical success of the CHR-P paradigm is determined by the concurrent integration of three core components: i) efficient detection of cases at-risk, ii) accurate prognosis, and iii) effective preventive treatment (18, 19). On a real-world scenario, these three components are developed, integrated, and monitored by the specialized CHR-P clinics that operate in different health care systems worldwide. Despite their crucial relevance to the implementation of the CHR-P paradigm in clinical practice, research into CHR-P services is relatively scarce. For example, the characteristics of CHR-P services, their relationship with the specific catchment areas, the type of outreach implemented, the characteristics of the service users, the types of indicated primary prevention strategies adopted, and the outcomes are not fully addressed. This lack of knowledge limits the broader scalability of the CHR-P paradigm and the associated clinical guidelines. For example, in April 2016, NHS England implemented a new Access and Waiting Times-Standard for Early Intervention in psychosis (AWT EI Standard) to extend the prevention of psychosis across England. The Standard mandates an evidence-based nationwide detection and rapid treatment of CHR-P patients aged 14 to 35 years. Therefore, the NHS requires all suspected patients presenting to early intervention services in England to be assessed and interviewed for a potential state of CHR-P (10). Yet, the standard does not regulate how CHR-P services should be best configured to achieve this goal. Similar lifespan preventive approaches are under consideration for development in other countries worldwide (20).

The current study aims at overcoming these limitations. We present the new consortium of CHR-P services that are active across London in the detection of individuals at-risk, their assessment and prognosis and their clinical management. The main aim was to describe the characteristics of CHR-P services across London, local catchment areas, outreach implemented, service users, indicated primary prevention strategies and outcomes. This information was then used to appraise the potential and clinical impact of the consortium for advancing the care of CHR-P individuals.

## Methods

This manuscript originates from a reflective workshop on the progress, challenges, and future directions of CHR-P services in the London area, which was held on November 21, 2018, at the Institute of Psychiatry, Psychology, and Neurosciences (IoPPN) at King’s College London. Senior representatives from a variety of CHR-P services attended the workshop. Following the conference, a proforma was created listing critical data to be collected for each CHR-P team, which included the following domains: i) description of the CHR-P service and catchment area, ii) outreach, iii description of service users, iv) interventions, and v) outcomes. When available, quantitative data were summarized in a descriptive table using mean and SD for continuous variables and frequencies for categorical variables. Furthermore, for each CHR-P service, we collected the initial sample size, the individual follow-up time, and the event (onset of psychosis). The current (2018) local population was estimated in each borough through the london.gov.uk website. We reported both total population and the 16- to 35-year age group, to better match the age range of the CHR-P patients. The local, as well as the national incidence of psychosis for the age range of 16 to 35 years, was estimated for each borough using PsyMaptic (http://www.psymaptic.org). The onset of psychosis was determined through the CHR-P instruments employed in each service. These data were then plotted and analyzed with a failure function (1-Kaplan Meier) and confidence intervals. The Kaplan Meier analysis was truncated when less than 10 individuals at-risk were available. The analyses were done in STATA.

## Results

### OASIS Lambeth and Southwark (OASIS)

#### Service and Catchment Area Description

**Figure 1:**
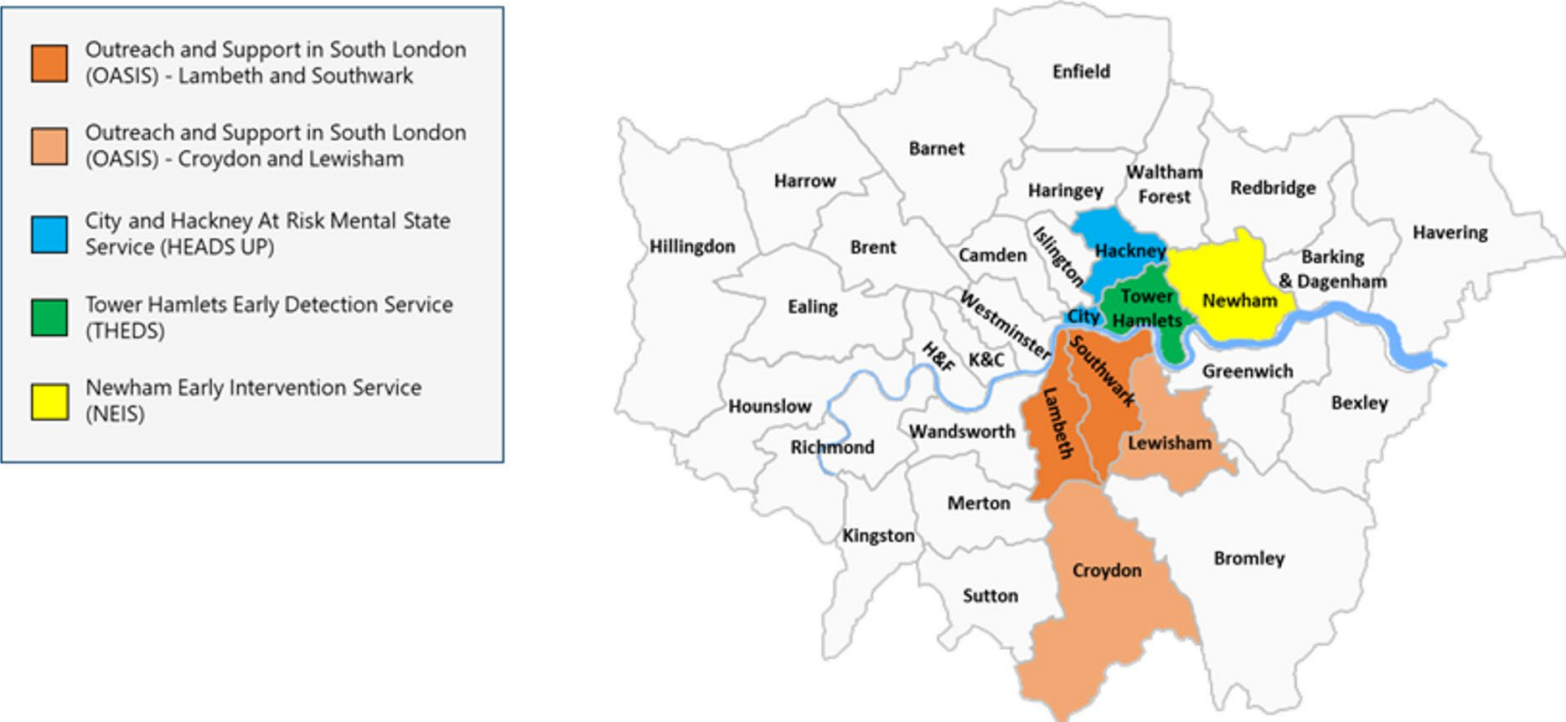
Pan-London Network for Psychosis-Prevention (PNP). Luton and Bedfordshire services were not included. K&C, Kensington and Chelsea; H&F Hammersmith and Fulham.

Outreach and Support In South-London (OASIS) was set up in 2001, and it is one of the oldest CHR-P services in the UK. The first 10 years of the service, along with its core characteristics have been presented in a previous publication (21). OASIS is part of the South London NHS Foundation Trust (SLaM) and provides early detection, assessment, and care for the boroughs of Lambeth (total population, 334,724; population age, 16–35 years; 133,543 in 2018 (22), **Figure 1**) and Southwark (total population, 322,302; population age, 16–35 years; 120,948 in 2018 (22), **Figure 1**). OASIS is a standalone service which is separated from the local first episode services.

**Figure 2:**
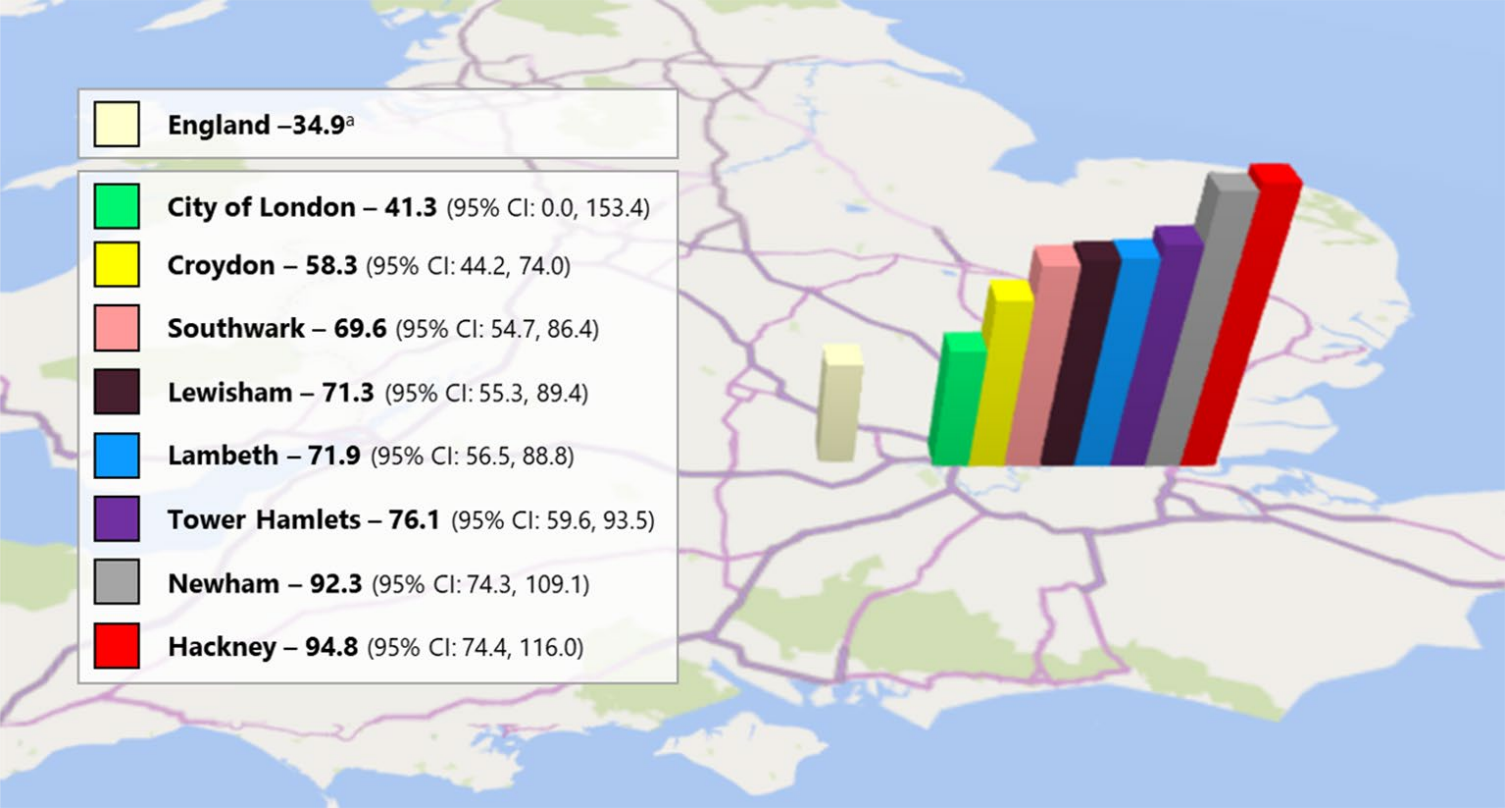
Pan-London crude predicted incidence of psychosis per 100,000 person-year (16-35 years). ^a^National average calculated from the PsyMaptic Psychosis LAD v1-0 xlsx file available at: http://www.psymaptic.org/prediction/psychosis-incidence-data/.

Incidence of psychosis in Lambeth and Southwark is estimated at 71.9 and 69.6 cases per 100,000 person-years, respectively, which is higher than England national average of 34.9 cases (**Figure 2**).

OASIS is closely linked with the Institute of Psychiatry, Psychology, and Neuroscience at King’s College London, and represents a successful integration of clinical research in the local NHS Trust.

The team is composed of two part-time consultant psychiatrists, a team leader, two clinical psychologists, an occupational therapist, a higher trainee psychiatrist, a part-time assistant psychologist, and a mental health nurse. In addition, there are several honorary visiting clinicians from all over the world who are supporting the clinical activities of the team. Inclusion criteria are being aged 14 to 35 years, having the general practitioner (GP) in the local borough, being help-seeking, and meeting the CHR-P criteria [determined with the Comprehensive Assessment of at Risk Mental States (CAARMS) 12/2006 (23)]. After inclusion OASIS provides care for 2-years. The caseload is approximately 80 patients per year.

#### Outreach

The success of OASIS is grounded on a long-standing and comprehensive outreach campaign with several local agencies. Details on the engagement activities of OASIS have been presented in previous publications (21, 24). GPs are the main source of referral to OASIS but referrals from self, caregivers or relatives, schools and colleges, social services or supported accommodations, community mental health services, inpatient mental health services, child and adolescent mental health services, early intervention for psychosis services, accident and emergency departments, police and criminal justice system, and physical health services are also allowed. More recently, a specific website has been launched to promote the clinical service (https://www.meandmymind.nhs.uk). 

#### Service User Description

Over the past years, the OASIS has taken care of 419 patients meeting the CAARMS 12/2006 (23) CHR-P criteria. The vast majority of service users met the Attenuated Psychosis Symptom (APS) subgroup of the CHR-P (**Table 1**), but Brief and Limited Intermittent Psychotic Symptoms (BLIPS) were also well represented (19.59%). Service users are mostly white (48.55%) young (age, 22.84 years) males (54.65%) who are single (78.48%) and unemployed (57.87%) at the time of contact with the service (**Table 1**). The presence of comorbid substance use or other psychiatric conditions is not an *a priori* exclusion criterion for OASIS and has been detailed in previous publications (25).

**Table 1:** Sociodemographic characteristics of the PNP.

		OASIS Lambeth and Southwark	OASIS Croydon and Lewisham	THEDS	HEADS UP	NEIS
Sample size		419	159	104	31	36
CHR-P assessment		CAARMS	CAARMS	SIPS	CAARMS	CAARMS
Yearly caseload		80	55	50	25	10
Age mean (SD)		22.84 (4.93)	22.01 (4.76)	22.02 (4.02)	23.35 (4.88)	22.33 (3.66)
Age range		14-35	13-36	17-26	17-33	18-31
Gender						
	% males	54.65	57.14	69.23	58.06	69.44
	% females	45.35	42.86	30.77	41.94	30.56
Ethnicity						
	White	48.55	40.76	23.11	58.06	22.22
	Asian	6.04	10.19	59.24	9.68	52.78
	Black African	9.42	6.37	8.20	9.68	0
	Black Caribbean	6.76	6.37	1.56	9.68	0
	Black British	16.43	19.75	0	0	25.00
	Other	12.8	16.56	7.89	12.9	0
Employment						
	% unemployed	57.87	68.59	51.25	29.03	75.00
	% students or employed	42.13	31.41	48.75	70.97	25.00
Marital status						
	% married/with a partner	21.52	16.13	6.5	22.58	5.56
	% single	78.48	83.87	93.5	77.42	94.44
Any substance misuse						
	% yes	NA	NA	40.00	45.16	27.78
	% no	NA	NA	60.00	54.84	72.22
CHR-P subgroup						
	% APS	80	75.72	NA	51.61	55.56
	% BLIPS/BIPS	19.59	19.65	NA	32.26	25.00
	% GRD	0.41	4.62	NA	16.13	19.44

Additional 38 CHR-P individuals were detected in the Luton and Bedfordshire services; CAARMS, comprehensive assessment of at-risk mental states;

SIPS, structured interview for psychosis-risk syndromes; APS, attenuated psychosis syndrome; BLIPS, brief and limited intermittent psychotic symptoms;

BIPS, brief intermittent psychotic symptoms; GRD, genetic risk and deterioration syndrome; CHR-P, clinical high risk for psychosis.

#### Interventions and Outcomes

As previously detailed, of the initial sample collected since inception, 33% of OASIS patients are treated with cognitive behavioral therapy (CBT) only; 17% of subjects received antipsychotics in addition to CBT sessions (26). Another 17% of subjects were prescribed with antidepressants in addition to CBT and 20% were exposed to a combination of interventions. The CBT + antidepressant intervention was associated with a reduced risk of transition to psychosis, as compared with the CBT + antipsychotic intervention (hazards ratio = 0.129) (26). Among CHR-P who will not develop psychosis, 28.3% still reported APS and 45.3% remain functionally impaired at follow-up (GAF <60) (27). A substantial proportion of patients (56.8%) is affected by at least one comorbid disorder at follow-up (27). Among CHR-P patients who presented with some comorbid disorder at baseline, 61.5% had persistent or recurrent course (27). Incident comorbid disorders emerged in 45.4% of baseline CHR-P patients (27). There was no increased risk of developing incident mental disorders in OASIS patients meeting CHR-P criteria compared with control groups (28). Underage patients remain under the care of children and adolescent mental health teams, but OASIS provides specialized care.

### OASIS Lewisham and Croydon (OASIS)

#### Service and Catchment Area Description

In 2014 to 2015, OASIS has expanded in two additional SLaM boroughs of Lewisham (total population, 310,324; population age, 16–35 years; 98,698 in 2018 (22), **Figure 1**) and Croydon (total population, 391,296; population age, 16–35 years; 101,336 in 2018 (22), **Figure 1**). Incidence of psychosis in Lewisham and Croydon is estimated at 71.3 and 58.3 cases per 100,000 person-years, respectively, which is higher than England national average of 34.9 cases (**Figure 2**).

The team is composed of two part-time consultant psychiatrists, a team leader, two clinical psychologists, a social worker, and a mental health nurse. Inclusion criteria, outreach, interventions, and outcomes have all been harmonized with OASIS Lambeth and Southwark and are presented in the section above. OASIS in Lewisham and Croydon is also a standalone service which is separated from the local first-episode services. The caseload is approximately 55 patients per year.

#### Service User Description

Over the past years, the OASIS has taken care of 159 patients meeting the CAARMS 12/2006 (23) CHR-P criteria. Most service users met the APS of the CHR-P intake criteria, but the BLIPS subgroup was also consistent (19.65%). Service users are mostly white (40.76%) young (age, 22.01 years) males (57.14%) who are single (83.87%) and unemployed (68.59%) at the time of contact with the service.

### Tower Hamlets Early Detection Service (THEDS)

#### Service and Catchment Area Description

The THEDS was fully operating from January 2010. It is part of the East London NHS Foundation Trust (ELFT), and it provides mental health promotion, early detection, and support in the Borough of Tower Hamlets (total population, 317,203; population aged 16 to 35 years; 135,832 in 2018 (22), **Figure 1**). Tower Hamlets has the fourth youngest population in the UK (29). Incidence of psychosis in Tower Hamlet is estimated at 76.1 cases per 100,000 person-year, surpassing the England national average of 34.9 cases (**Figure 2**).

The team is composed of one team leader (nurse), a part-time senior practitioner, a part-time clinical psychologist, and one day of a consultant psychiatrist. THEDS is like OASIS, one of the few standalone services of this kind; nevertheless, it works closely with the Early Intervention Service and shares premises with them.

Inclusion criteria to THEDS are: 16 to 25 years, having the GP in the local borough, being help-seeking and meeting the CHR-P criteria [Structured Interview for Psychosis-risk Syndromes (SIPS) (30)]. After inclusion, THEDS offers 2 years of follow-up and support to service users and carers. The caseload fluctuates between 30 and 50 every given year.

#### Outreach

From the beginning, THEDS had a strong focus on outreach and mental health promotion. The team has established a close relationship with community-based services; it is present in the community and educational events, it meets with GPs regularly and trains front-line youth workers offering on-site presentations, consultation, and feedback over the phone. We also train new doctors and focus on changing policies around youth mental health in Tower Hamlets. THEDS has also developed a website (https://theds.elft.nhs.uk/) and is present in social media: facebook and twitter. We have included our young service users in their development. THEDS accepts referrals from GP (21%), secondary care (20%), primary care psychology (13%), third sector organizations (11%), self-referrals (14%), education (9%), and from carers. Every person that is assessed will receive a mental health promotion package and will be signposted or referred to the adequate service for them if they are not meeting CHR-P criteria.

#### Service User Description

About one third of the patients assessed met the CHR-P criteria. Most of them met the APS subgroup. Service users are mostly Asian (59.24%) young (age 22.02%) males (69.23%), single (93.5%) and unemployed (51.25%). Among service users, mood disorders are the most prevalent comorbid condition (56% of total comorbidities), followed by substance misuse (10%), and anxiety disorders (7%). Service users with CHR-P symptoms potentially secondary to other factors (e.g. substance use) are admitted to the service and offer a new assessment after 6 months (*extended assessment*). In the meantime, they are offered the same package of care as standard service users. Self-reported use of substances among the CHR-P population was 40%, cannabis is the most common substance used (28%). THEDS works closely with local substance misuse services and occupational services in the borough.

#### Interventions and Outcomes

THEDS offers 2 years of follow-up and mental state monitoring, casework and intervention. THEDS focuses on psychoeducation and psychosocial support. Every client is case worked, and support on job retention/seeking, education, and social needs is offered. THEDS also provides CBT (accepted by 65% of patients) and brief family interventions (accepted by 30% of patients) routinely. THEDS offers medical reviews and psychopharmacological treatment. Medication is mostly used to treat affective comorbidities. THEDS may prescribe low-dose antipsychotics when the symptoms interfere with functionality.

### City & Hackney At-Risk Mental State Service (HEADS UP)

#### Service and Catchment Area Description

The City & Hackney At-Risk Mental State Service (HEADS UP) has been in operation since 2015, initially, as a 1-year pilot which was then made permanent. It is part of the ELFT, providing early detection and support services in the City of London (total population 7,681; population aged 16–35 2,530 in 2018 (22), **Figure 1**) and Hackney boroughs [total population, 281,740; population age, 16–35 years; 105,945 in 2018 (22)]. Incidence of psychosis in the City is 41.3 and Hackney 94.8 cases per 100,000 person-year. Incidence of psychosis in Hackney is over twice the England national average of 34.9 cases and is one of the highest in the whole of the country (**Figure 2**).

The team is composed of one part-time team leader, one senior nurse practitioner, one part-time clinical psychologist, and two part-time consultant psychiatrists. HEADS UP is an embedded service, sharing human and financial resources with the local first episode early intervention service (EQUIP).

Inclusion criteria to HEADS UP are: 18 to 35 years of age, having the GP in the local boroughs, being help-seeking, and meeting CHR-P criteria [CAARMS 12/2006 (23)]. After inclusion, HEADS UP provides 2-year support to service users and carers. The caseload is capped at 25 service-users at any given time, with the intention to expand in the future.

#### Outreach

Outreach and service promotion is conducted *via* visits and training activities in GP offices and mental health services across ELFT. GPs are the primary source of referrals to HEADS UP (61%).

#### Service User Description

Between October 2015 and October 2017, 31 individuals had met criteria for CHR-P, mostly APS (50%) but the BLIPS subgroup is highly frequent (33%). Service users are mostly white (58.06%) young (age 23.4 years) males (58%), single (77%, **Table 1**). Most of them are either employed (45%) or in education (26%, **Table 1**). Any type of substance misuse is present in almost half (45%) of service users. Cannabis is the most common substance. The presence of comorbid substance use or other psychiatric conditions is not an *a priori* exclusion criteria for HEADS UP unless these conditions are assessed as the primary cause in the clinical presentation.

#### Interventions and Outcomes

All service users have a key worker providing psychosocial support and ongoing monitoring of the mental state. Most service users (77%) have received CBT-informed individual psychotherapy, and less frequently (19%), family therapy. Other psychosocial interventions include psychoeducation and practical support (education, housing, work). Medical reviews and psychopharmacological treatment are also offered. About half of service users receive psychopharmacological treatment, with antidepressants being the most commonly prescribed drugs. Only 1 of 31 service users has received low-dose antipsychotic medication.

### Newham Early Intervention Service (NEIS)

#### Service and Catchment Area Description

The NEIS was established in 2008, with the aim of providing mental health services to young people at increased risk of psychosis and those who experience a first episode of psychosis. It is part of the ELFT and operates in the London borough of Newham (population, 353,245; population age, 16–35 years; 132,057 in 2018 (22), **Figure 1**). Incidence of psychosis in Newham is over twice the England national average of 34.9, with 92.3 cases per 100,000 person-year (**Figure 2**). An integrated model is in place at NEIS, and the CHR-P team shares financial and human resources with the first-episode team. The CHR-P team is not separate. The staff within the NEIS provides input to both first-episode psychosis patients and those at CHR-P. Inclusion criteria are: 18 to 35 years of age, having the GP in the local borough, meeting CHR-P criteria [CAARMS 12/2006 (23)], and being help-seeking. After inclusion, NEIS provides up to 3-year support to CHR-P service users. The caseload of CHR-P service-users is around 10 at any given time and is not capped. 

#### Outreach

An assertive outreach strategy is conducted by the NEIS, mostly directed at GP training on CHR-P symptoms. Intentions to expand community outreach activities to incorporate third-party and education organizations are currently constrained by limited financial and human resources. Overall, engagement of CHR-P service users is better than that in the first-episode service although referral rates are lower.

#### Service User Description

Up to 36 individuals met CHR-P criteria, mostly the APS subgroup (55.56%). The BLIPS subgroup was also quite well represented (25%). Service users were mostly Asian (52.78%), unemployed (75%), single (94.44%), and of young (age 22.33) males (69.44%). About one third (27.78%) were presenting with any substance use. Comorbid conditions are not an exclusion criterion to be eligible for the service.

#### Interventions and Outcomes

CHR-P service users are offered medical and psychosocial support and ongoing mental state monitoring. Each service user has monthly sessions with a health care professional. In addition, available psychosocial interventions include CBT-informed individual psychotherapy, family therapy, and needs-based support. Low-dose antipsychotic medication is used in a small number of service users.

### Risk of Psychosis Across the PNP Network

**Figure 3:**
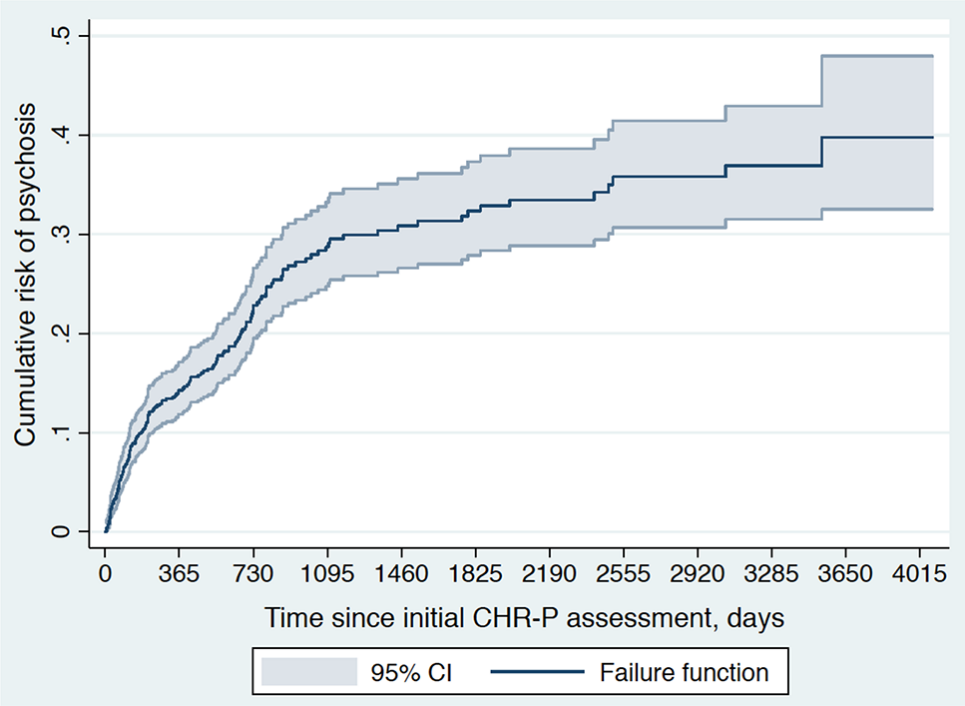
Cumulative risk of psychosis onset (failure function) in 787 CHR-P individuals from the Pan-London Network for Psychosis-Prevention (PNP). There were 503 individuals at-risk at year 1, 283 individuals at-risk at year 2, 192 individuals at-risk at year 3, 158 individuals at-risk at year 4, 139 individuals at-risk at year 5, 113 individuals at-risk at year 6, 110 individuals at-risk at year 7, 72 individuals at-risk at year 8, 42 individuals at-risk at year 9, and 29 individuals at-risk at year 10. The function was truncated at 4081 days of follow-up when 10 individuals were still at-risk for psychosis.

A total of 787 individuals at-risk for psychosis were followed up by the PNP (including 38 individuals from the Bedforshire and Luton service). The risk for psychosis onset was: 0.141 (95% CI, 0.117–0.169) at 1 year, 0.221 (95% CI, 0.189–0.258; 503 individuals still at-risk) at 2 years, 0.281 (95% CI, 0.242–0.325; 283 individuals still at-risk) at 3 years, and 0.301 (95% CI, 0.259–0.347; 158 individuals still at-risk) at 4 years (**Figure 3**).

### Translational Clinical Research in the PNP

There was consensus across PNP teams that improving the detection of individuals at-risk was the most challenging operational challenge to be overcome. A complementary strategy targeting secondary mental health care, primary care, and the community was discussed to improve the recruitment of CHR-P individuals. Accordingly, some PNP teams have set up new websites to facilitate detection of CHR-P individuals from the community. The PNP is also committed to supporting some research innovations that can improve detection of CHR-P individuals in secondary mental health care. These involve the use of transdiagnostic risk calculators to screen electronic health records and identify potential candidates for CHR-P assessment (19, 31, 32) as part of MRC-funded grant (MC_PC_16048). Other research innovations conducted in the PNP involve the development of personalized prediction algorithms (33) as part of large-scale international consortia (e.g., PSYSCAN, http://psyscan.eu) or experimental therapeutics studies in CHR-P individuals [e.g., intranasal oxytocin (34, 35)].

## Discussion

The PNP included five CHR-P services across two NHS Trusts: Outreach and Support In South-London (OASIS) in Lambeth and Southwark, OASIS in Croydon and Lewisham, Tower Hamlets Early Detection Service (THEDS), City & Hackney At-Risk Mental State Service (HEADS UP) and Newham Early Intervention Service (NEIS). The PNP serves a total population of 2,318,515 Londoners (830,889; age, 16–35 years), with a yearly recruitment capacity of 220 CHR-P individuals (age, 22.55 years). Standalone teams (OASIS and THEDS) are more established and successful than teams that share their resources with other mental health services (HEADS UP, NEIS). Characteristics of the catchment areas, outreach, and service users, differ across PNP services; all of them offer psychotherapy to prevent psychosis. The PNP is supporting several CHR-P translational research projects.

This is the first network of CHR-P services to be established in the UK. It serves an extensive and diverse urban population of 2,318,515 Londoners (830,889; age, 16–35 years), which represent the largest catchment area for the recruitment of CHR-P individuals. Correspondingly, the sociodemographic characteristics of the young people meeting CHR-P criteria were highly heterogeneous across PNP’s teams. First, the incidence of psychosis in the PNP was higher than in the rest of the country, likely reflecting the impact that the urban environment exerts on the onset of this disorder (36). Second, CHR-P patients under the care of the PNP were mostly unemployed males living alone. Third, non-white ethnic minorities were highly represented across users of CHR-P services. This is particularly important in light of the patterns of ethnic inequalities in pathways to psychiatric care, particularly affecting black groups (37), because it confirms that CHR-P services can provide easier access to care to ethnic minorities (16). The age limit of PNP service users ranged from 14 to 35 years, and the average age of CHR-P individuals was of 22.55 years. Therefore, the PNP teams have disregarded the recommendation of the Access and Waiting Time Standard, which has extended to the age 65 the acceptance criteria for the majority of early intervention in psychosis (and CHR-P) services in the UK (38). The Standard’s recommendation is not supported by any psychometric validity because the CHR-P instruments have been validated in mostly in the age range of 14 to 35 years with possibilities to extend it to 8 to 40 years (7). There is not even epidemiological support for the Standard’s recommendation: psychotic disorders, despite being relatively rare before the age of 14 years (39); peak in the age group of 15 to 35 years, and declines after the age of 35 years (36). Another recent appraisal of the standard confirmed that it is unclear how adopting the recommended extended age range and treating CHR-P cases could impact on the positive outcomes already associated with 14 to 35 years age range (40). A further independent survey of CHR-P services in the UK concluded that current provision for CHR-P in England does not match clinical guidelines (41). Fourth, concerning illicit substance misuse, only a minority of CHR-P individuals reported using them. The actual impact of illicit substance use, such as cannabis, on the risk of transitioning to psychosis from a CHR-P stage is not wholly clear (42). Fifth, with respect to other characteristics of the PNP service users, data were scattered and not always reported. Collecting real-world clinical data is not straightforward outside the research setting. This task could be facilitated by the availability of a standard data acquisition platform that incorporates the core CHR-P measures and that can be used in clinical routine; the proforma developed for this study can serve as the starting platform. The development of a core CHR-P measurements package to be consistently used Pan-London will be the subject of future PNP work. At the same time, the methods employed for this study, which represent the largest appraisal of CHR-P services in London, could be employed to conduct a national deep dive surveying the implementation level, operational policies and challenges of CHR-P services in the UK. New national infrastructures, such as the National Institute of Health Research—Mental Health Translational Research Collaboration Early Psychosis Workstream (https://www.nihr.ac.uk/news/the-uks-leading-mental-health-experts-unite-to-solve-treatment-challenges/9193) could tackle this task.

In terms of outreach campaigns, there was high variability across PNP’s teams. The problem of heterogeneous recruitment strategies for CHR-P patients and their profound impact on the level of risk enrichment that is eventually observed have been fully addressed in previous publications by our group [see Refs. (24, 43–46)]. Once the CHR-P individuals were recruited into the PNP, they were assessed with the CAARMS or with the SIPS. This operational approach diverges from the National Institute of Clinical Excellence (NICE) recommendations which suggest using the CAARMS only. However, there are no diagnostic or prognostic advantages of any psychometric interview over each other (10) (the SIPS has slightly superior sensitivity than the CAARMS (47). Furthermore, recent automatic packages allow the scores of the two interviews to be reciprocally converted (6). Therefore, current clinical guidelines should consider a more flexible approach to allow the implementation of the CHR-P paradigm. Another critical issue related to the presence of comorbid mental disorders, all the PNP teams allowed their concurrent presence in addition to CHR-P criteria, in line with established literature (25).

The PNP’s clinical potentials cumulate in one of the largest real-world clinical cohort of CHR-P individuals worldwide, encompassing 787 individuals and a 4-year follow-up. In the PNP about one (30%) in three CHR-P individuals developed a psychotic disorder within 4 years. This value suggests that the actual transition risk of clinical cohorts, as opposed to research cohorts may not be declining. Such an effect could be to sampling biases, with more severe patients declining participation in research studies (48). The PNP’s risk was observed in the context of naturalistic design with the potential confounding effect of preventive interventions such as CBT. However, the actual effect of preventive treatments for the reduction of the risk of psychosis onset is unclear (14). Clinical guidelines (e.g., NICE) are not updated to reflect the current status of knowledge, with respect to effective treatments for CHR-P patients. Of interest, there was a relatively high proportion of CHR-P individuals meeting the short-lived intake criterion (Brief and Limited Intermittent Psychotic Symptoms [BLIPS (49)], which may reflect the high incidence of psychosis in the PNP. Overall, the risk estimates of transition to psychosis can be used as a national benchmark for other future CHR-P studies in the UK. Notably, the PNP has an extensive recruitment capacity which cumulates to 220 individuals per year. This opens the door to the large-scale implementation of clinical research innovations in the London area, potentially impacting the lives of many young people. As noted above, the PNP has already implemented innovative approaches for improving the detection of CHR-P individuals in the community and secondary mental health care, to produce a personalized prediction of their outcomes and to test first-in-class experimental therapeutics. These studies have demonstrated the ability of the PNP to achieve an optimal integration of clinical and research aspects, which is pivotal to the successful translation of research innovations in clinical routine. Since the PNP leverages a universal healthcare system and NHS infrastructure, it offers competitive advantages compared to other international CHR-P infrastructures that are characterized by heterogeneous clinical scenarios.

A final important operational issue related to the configuration of CHR-P services in the PNP. OASIS and THEDS were standalone services, separated from the local early intervention services in terms of staffing resources and team leadership (they only shared the premises). Conversely, HEADS UP and NEIS were CHR-P services embedded within the local early intervention services, sharing staff, and team leadership. Standalone services appeared more successful than embedded services on several implementations, delivery and outcome measures. For example, **Table 1** clearly reports higher yearly caseloads for standalone teams compared with services that are embedded within first-episode services. This could be due to the fact that the CHR-P and first episode populations are different in terms of clinical needs. Although patients experiencing a first episode of psychosis typically are more acute and severe, CHR-P individuals require more subtle assessment and follow-up. Embedding CHR-P services within early intervention services may end up penalizing the less severe patients because the staff would tend to invest more time and effort in taking care of those more unwell. There was consensus across the PNP that standalone CHR-P services are more efficient than embedded CHR-P services in terms conducting the outreach, initial assessment, delivery or psychological therapies, and longitudinal follow-up. In line with these findings, the first appraisal of the Access and Awaiting Time Standard confirmed these operational issues and the importance of clear treatment pathways and targeted interventions that would need to develop and commission of distinct and standalone CHR-P services (40). Another independent UK survey has evidenced that only 42% to 50% of the CHR-P services that are embedded within early intervention services are in reality able to offer the NICE recommended treatments: CBT, family intervention, and training on CBT (41). Against the current clinical guidelines, 50% of embedded CHR-P services used antipsychotic treatments (41). Implementing dedicated CBT for CHR-P individuals in clinical routine is demanding in terms of staffing, training, and financial resources; this is hardly achieved in the context of first-episode teams that are already stretched with the provision of the same treatments for more severe and acute patients. Only standalone CHR-P services can ensure that sufficient time, financial resources, training, and clinical knowledge is devolved to the detection, prognostic assessment, and clinical care of CHR-P patients. This study concluded that this is because the majority of early intervention services in England did not receive allocated funding for the CHR-P population, and there are few standalone services specifically commissioned for these patients (41).

Limitations of this study included the lack of a common data acquisition dataset to record service-related data that could characterize CHR-P services. For example, the proportion of patients exposed to different types of preventive treatments was not consistently recorded across the different PNP services. The development of a standardized data acquisition system across CHR-P services will be the core objective of the next stages of the PNP. This could then be scaled up nationally and further benefit CHR-P services outside London.

In conclusion, this is the largest survey of CHR-P services in the UK, which focuses on a Pan-London area. The PNP represents the largest NHS collaboration across CHR-P services in the UK. The appraisal of the PNP can have some impact on the field. First, the extension of the CHR-P assessment to those older than 35 to 40 years is not justified. Rather, it may be possible to lower the age range to 12 years (50) to better capture young at-risk populations. Second, it is essential to develop a standard data acquisition platform and a package of measures to be recorded by CHR-P services in the UK to facilitate the appraisal of their success and the associated challenges. The next step would involve conducting a national survey. Third, NICE guidelines should be updated to allow the use of different CHR-P instruments and to recognize the challenges that are currently associated with the use of psychological therapies to prevent psychosis in this population. Fourth, the PNP represents a benchmark infrastructure to conduct translational research in the UK and could be considered for future national initiatives. Fifth, standalone CHR-P teams should be recommended by the national clinical guidelines, and their development should be supported by adequate funding. Overall, this study highlights crucial operational issues which will need careful consideration in the future planning of CHR-P services.

## Conclusion

The PNP is the largest CHR-P clinical network in the UK; it represents a reference benchmark for implementing detection, prognosis, and care in the real-world clinical routine, as well as for translating research innovations into practice.

## Data Availability Statement

The datasets generated for this study will not be made publicly available. There is no ethical permission for data sharing.

## Ethics Statement

The study received ethical approval as an audit by the local NHS Trusts.

## Author Contributions

PF-P conceived the study. AE conducted it along with TS, SG, SM-A, SE, KW, OA, JB, MS, SF, VS, AA, PM and AM. PF-P provided ongoing supervision. PF-P drafted the first version of the manuscript and all authors contributed to the interpretation of findings.

## Funding

This study was supported by the King’s College London Confidence in Concept award from the Medical Research Council (MRC) (MC_PC_16048) to PFP. These funding bodies had no role in the design of the study, collection, and analyses.

## Conflict of Interest

The authors declare that the research was conducted in the absence of any commercial or financial relationships that could be construed as a potential conflict of interest.
